# Kalman contrastive unsupervised representation learning

**DOI:** 10.1038/s41598-024-76085-7

**Published:** 2024-12-04

**Authors:** Mohammad Mahdi Jahani Yekta

**Affiliations:** Department of Computer Science, Stanford University, 353 Jane Stanford Way, Stanford, CA 94305 USA

**Keywords:** Contrastive unsupervised learning, Dictionary building, Kalman filter, MoCo, Regularized optimization, Applied mathematics, Computer science

## Abstract

We first propose a Kalman contrastive (KalCo) framework for unsupervised representation learning by dictionary lookup. It builds a dynamic dictionary of encoded representation keys with a queue and a Kalman filter encoder, to which the encoded queries are matched. The large and consistent dictionaries built this way increase the accuracy of KalCo to values much higher than those of the famous momentum contrastive (MoCo) unsupervised learning, which is actually a very simplified version of KalCo with only a fixed scaler momentum coefficient. For a standard pretext task of instance discrimination on the ImageNet-1M (IN-1M) dataset; e.g., KalCo yields an accuracy of 80%, compared to 55% for MoCo. Similar results are obtained also on Instagram-1B (IG–1B). For the same task on a bunch of OpenfMRI datasets, the accuracy is 84%. We then upgrade KalCo to KalCo v2 by using an MLP projection head and more data augmentation, along also with a larger memory bank. The accuracy of KalCo v2 is around the even more impressive amounts of 90% on IN-1M and IG-1B, and 95% on OpenfMRI, the first being about 3% higher than those of three most-cited recent alternatives.

## Introduction

**Fig. 1:**
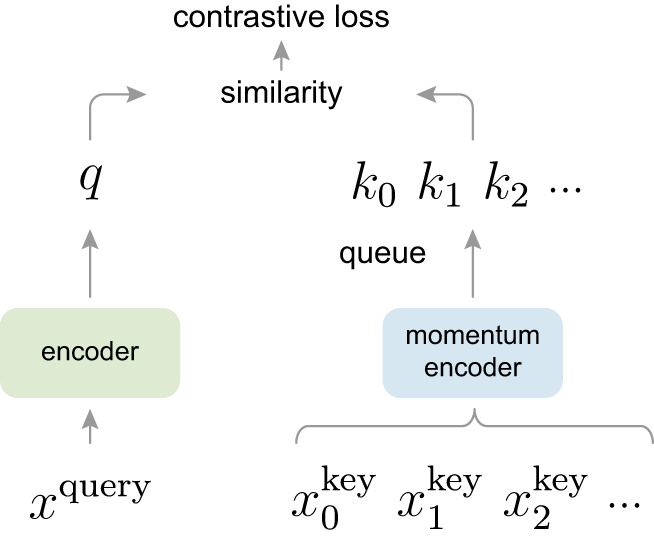
Momentum contrast (MoCo, source: 1).

MoCo version 1 (v1)^1^, with 13.7k+ Google Scholar citations as of Dec. 2024, is a way of building large and consistent dictionaries for unsupervised learning with a contrastive loss (Fig. 1). It trains a visual representation encoder by matching an encoded query *q* to a dictionary of encoded keys using a contrastive loss.

With similarity measured by dot product, the form of contrastive loss function used in^1^ is InfoNCE^2^, defined as

1 $$\begin{aligned} \mathcal {L}_{q, k^+, \{k^-\}} = -\log \frac{\exp (q{\cdot }k^+ / \tau )}{\exp (q{\cdot }k^+ / \tau ) + {\displaystyle \sum _{k^-}}\exp (q{\cdot }k^- / \tau )}. \end{aligned}$$

Here $k^+$ is a representation of the positive (similar) key sample, and $\{k^-\}$ are representations of the negative (dissimilar) key samples. $\tau$ is a temperature hyper-parameter^3^. In the *instance discrimination* pretext task^3^ used by MoCo and SimCLR^4^, a query and a key form a positive pair if they are data-augmented versions of the same image, and a negative pair otherwise. 

The contrastive loss serves as an unsupervised objective function for training the encoder networks that represent the queries and keys. In general, the query representation is $q=f_\text {q}(x^q)$, where $f_\text {q}$ is an encoder network and $x^q$ is a query sample (likewise, $k=f_\text {k}(x^k)$). Their instantiations depend on the specific pretext task. The networks $f_\text {q}$ and $f_\text {k}$ can be identical^5^, partially shared^2^, or different^6^.

**Fig. 2:**
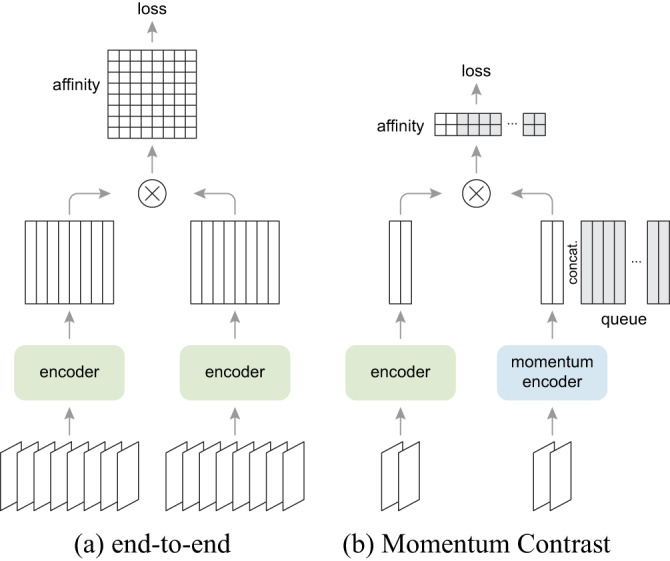
A batching perspective of two optimization mechanisms for contrastive learning. Images are encoded into a representation space, in which pairwise affinities are computed (source: 7).

The dictionary keys $\{k_0, k_1, k_2,...\}$ are defined on-the-fly by a set of data samples. The dictionary is built as a queue, with the current mini-batch enqueued and the oldest mini-batch dequeued, decoupling it; in contrast to the end-to-end mechanism^2^, from the mini-batch size (Fig. 2)^7^. The keys are encoded by a slowly progressing encoder, driven by a momentum update along with the query encoder. This method enables a large and consistent dictionary for learning visual representations^1^.

Denoting the parameters of $f_\text {k}$ as $\varvec{\uptheta }_\text {k}$ and those of $f_\text {q}$ as $\varvec{\uptheta }_\text {q}$, the momentum update rule for dictionary-building, proposed heuristically in^1^, is

2 $$\begin{aligned} \hat{\varvec{\uptheta }}_\text {k}(n+1)=m\hat{\varvec{\uptheta }}_\text {k}(n)+(1-m)\varvec{\uptheta }_\text {q}(n+1), \end{aligned}$$

with *m* being a momentum coefficient close to 1, suggested there; again heuristically, to be around 0.999. The above paper does not present any theory neither for the mechanics of this formula, nor for what the optimal *m* is.

**Our contributions.** In this paper we propose a Kalman filter updating for $\hat{\varvec{\uptheta }}_\text {k}$s, which includes (2) as its very simplified version with only a fixed scaler momentum coefficient. Experiments show that the accuracy of KalCo can be up to 22–25% higher than that of MoCo. The KalCo framework also provides us with the dynamic optimal *m* for MoCo, which is set there heuristically and static. The accuracy of scaler KalCo (with dynamic optimal *m*) is also observed to be about 10–12% higher than that of MoCo. We then upgrade KalCo to KalCo v2 by using an MLP projection head and more data augmentation, along also with a larger memory bank. The accuracy of KalCo v2 is around the even more impressive amounts of 90% on IN-1M and IG-1B, and 95% on OpenfMRI, the first being about 3% higher than those of three most-cited recent alternatives.

## Kalman filter updating of the key parameters

Kalman filters, reviewed below shortly in the context of our usage of them, are nonlinear dynamical systems that have extensive applications in data processing. Here we propose to model the evolution of $\varvec{\uptheta }_\text {k}$ and $\varvec{\uptheta }_\text {q}$ in the form of a Kalman filter as

3a $$\begin{aligned} \varvec{\uptheta }_\text {k}(n)&=\textbf{F}(n)\varvec{\uptheta }_\text {k}(n-1)+\textbf{u}(n), \end{aligned}$$

3b $$\begin{aligned} \varvec{\uptheta }_\text {q}(n)&=\textbf{H}(n)\varvec{\uptheta }_\text {k}(n)+\textbf{v}(n). \end{aligned}$$

In the above,

1. Eq. (3a) governs the evolution of the internal state $\varvec{\uptheta }_\text {k}$ of the process, to which we don’t have direct access. We can observe it only through Eq. (3b), as hinted at in item (4) below.
2. $\textbf{F}(n)$ is the state transition matrix.
3. $\textbf{u}(n)$ is classically referred to as the process noise. This title should not however cause $\textbf{u}$ to be interpreted as a literal unwanted “noise” that exists in the system inevitably. It is instead in fact the unpredictable “innovation” or “residual” component of the internal state $\varvec{\uptheta }_\text {k}(n)$, which is independent of and orthogonal to $\varvec{\uptheta }_\text {k}(i)$ ($i=1, \ldots , n-1$); i.e., with $\mathbb {E}$ denoting statistical expected values, $\mathbb {E}\{\varvec{\uptheta }_\text {k}^\textrm{T}(i) \textbf{u}(n)\}=0$. In our context, it counts for the unpredictable modification that arrival of a new key sample makes on the parameters of the key encoder. It is assumed to be drawn from a zero mean multi-variable normal distribution $\mathcal {N}(\textbf{0}, \textbf{Q}(n))$.
4. Eq. (3b) describes the observation (or measurement) $\varvec{\uptheta }_\text {q}(n)$ of $\varvec{\uptheta }_\text {k}(n)$.
5. $\textbf{H}(n)$ is the observation model, which maps the internal state space into the observed space.
6. $\textbf{v}(n)$ is the observation noise, with the explanation of the term “noise” similar to that of $\textbf{u}(n)$. It is assumed to be drawn from a zero mean multi-variable normal distribution $\mathcal {N}(\textbf{0}, \textbf{R}(n))$.
7. The initial state $\varvec{\uptheta }_\text {k}(0)$ and the noise vectors at all instants ($\textbf{u}(1)$, ..., $\textbf{u}(n)$, $\textbf{v}(1)$, ..., $\textbf{v}(n)$) are all assumed to be mutually independent.

In theory, we may also assume that the matrices $\textbf{F}(n)$ and $\textbf{H}(n)$ change sufficiently slowly, and can thus be estimated once e.g. from the keys computed in the end-to-end mechanism. In practice, we of course estimate them via other approaches, to be explained later.

Kalman filter is in fact a recursive linear estimator that makes the best linear estimate of $\varvec{\uptheta }_\text {k}(n)$ given the observations $\varvec{\uptheta }_\text {q}(0)$ to $\varvec{\uptheta }_\text {q}(n)$, irrespective of the stochastic distributions of $\textbf{u}$ and $\textbf{v}$. For normal distributions of these variables, this linear estimate is the best Bayesian estimate as well. Its algorithm is presented below. We denote the involved vectors and matrices before and after the arrival of $\varvec{\uptheta }_\text {q}(n)$ by the − and + superscripts respectively. The covariance matrix of $\varvec{\uptheta }_\text {k}(n)$ is also shown by $\textbf{P}(n)$.

### The Kalman filter algorithm

Let $\varvec{\uptheta }_\text {k}(0)$ be a vector with mean $\varvec{\uptheta }_0$ and covariance matrix $\textbf{P}(0)$. For a deterministic $\varvec{\uptheta }_\text {k}(0)$, as we expect to have in our context, $\textbf{P}(0)=\textbf{0}$, but this is not a necessary assumption. Kalman filter initializes as:

**Initialization**

4a $$\begin{aligned} \hat{\varvec{\uptheta }}_\text {k}^-(0)&= \varvec{\uptheta }_0 \end{aligned}$$

4b $$\begin{aligned} \textbf{P}^-(0)&=\textbf{P}(0). \end{aligned}$$

It then updates the a posteriori (+) estimates of the involved variables at time *n* as:

**State update**

5a $$\begin{aligned} \hat{\varvec{\uptheta }}_\text {q}(n)&= \textbf{H}(n)\hat{\varvec{\uptheta }}_\text {k}^-(n) \end{aligned}$$

5b $$\begin{aligned} \textbf{K}(n)&= \textbf{P}^-(n)\textbf{H}^\textrm{T}(n)\left[ \textbf{H}(n)\textbf{P}^-(n)\textbf{H}^\textrm{T}(n)+\textbf{R}(n)\right] ^{-1} \end{aligned}$$

5c $$\begin{aligned} \hat{\varvec{\uptheta }}_\text {k}^+(n)&= \hat{\varvec{\uptheta }}_\text {k}^-(n)+\textbf{K}(n)\big (\varvec{\uptheta }_\text {q}(n)-\hat{\varvec{\uptheta }}_\text {q}(n)\big ) \end{aligned}$$

5d $$\begin{aligned} \textbf{P}^+(n)&= [\textbf{I}-\textbf{K}(n)\textbf{H}(n)]\textbf{P}^-(n). \end{aligned}$$

$\textbf{K}(n)$is referred to as the Kalman gain. The a priori (-) estimates of the variables, to be used at time $n+1$, are also computed as:

**State propagation**

6a $$\begin{aligned} \hat{\varvec{\uptheta }}_\text {k}^-(n+1)&= \textbf{F}(n+1)\hat{\varvec{\uptheta }}_\text {k}^+(n) \end{aligned}$$

6b $$\begin{aligned} \textbf{P}^-(n+1)&= \textbf{F}(n+1)\textbf{P}^+(n)\textbf{F}^\textrm{T}(n+1)+\textbf{Q}(n+1). \end{aligned}$$

### Proof outline

The above formulas can be derived by induction, taking into account the two equations

7a $$\begin{aligned}&\mathbb {E}\big \{\hat{\varvec{\uptheta }}_\text {k}^+(n+1)\big \}= \mathbb {E}\big \{\varvec{\uptheta }_\text {k}(n+1)\big \}, \end{aligned}$$

7b $$\begin{aligned}&\mathbb {E}\big \{(\varvec{\uptheta }_\text {k}(n+1)-\hat{\varvec{\uptheta }}_\text {k}^+(n+1))\varvec{\uptheta }_\text {q}(n+1)\big \}= 0, \end{aligned}$$

the latter classically known as the “orthogonality” principle. It is not of course actually a literal “principle”, and is derived by setting the derivative of the error $\big (\varvec{\uptheta }_\text {k}(n+1)-\hat{\varvec{\uptheta }}_\text {k}^+(n+1)\big )^2$ with respect to the coefficient of $\varvec{\uptheta }_\text {q}(n+1)$ equal to zero. Details can be found in any standard textbook on linear estimation.

## Estimation of the Kalman matrices

In the theoretical case, where all the Kalman coefficients and noise covariance matrices are available, we can use the above algorithm to update $\varvec{\uptheta }_\text {k}$ ideally as

8 $$\begin{aligned} \hat{\varvec{\uptheta }}_\text {k}(n+1)=\left[ \textbf{I}-\textbf{K}(n+1)\textbf{H}(n+1)\right] \textbf{F}(n+1)\hat{\varvec{\uptheta }}_\text {k}(n)+ \textbf{K}(n+1)\varvec{\uptheta }_\text {q}(n+1). \end{aligned}$$

In practice, however, we don’t have all or maybe even any of the above matrices explicitly available. This is because we assumed them to have been derive from the end-to-end mechanism, while we are now using KalCo. Nevertheless, if the changes of these elements would be slow enough for them to be modeled; over a sufficiently long range of *n*, with time-independent (and somehow inferable) counterparts $\mathbf{F}$ and $\mathbf{H},$ Eq. (8) can be simplified to

9 $$\begin{aligned} \hat{\varvec{\uptheta }}_\text {k}(n+1)=\left[ \textbf{I}-\textbf{K}(n+1)\textbf{H}\right] \textbf{F}\hat{\varvec{\uptheta }}_\text {k}(n)+ \textbf{K}(n+1)\varvec{\uptheta }_\text {q}(n+1). \end{aligned}$$

Note that $\textbf{K}$, $\textbf{P}^-$, and $\textbf{P}^+$ still depend on *n*. 

If no knowledge about the involved matrices are available, we may model them heuristically as $\textbf{F}(n), \textbf{H}(n)=\textbf{I},$ $\textbf{Q}(n)=q^2\textbf{I}$, and $\textbf{R}(n)=r^2\textbf{I}$, with $\textbf{I}$ being the identity matrix, and *q* and *r* scaler noise variances. For each batch, *q* and *r* can be inferred from those of the previous ones. With these, the Kalman parameters will converge after some time to the approximate values of

10a $$\begin{aligned} \textbf{K}&\simeq \frac{q}{r+q}\textbf{I}, \end{aligned}$$

10b $$\begin{aligned} \textbf{I}-\textbf{K}&\simeq \frac{r}{r+q}\textbf{I}, \end{aligned}$$

10c $$\begin{aligned} \textbf{P}^-&\simeq rq\textbf{I}, \end{aligned}$$

10d $$\begin{aligned} \textbf{P}^+&\simeq \frac{r^2q}{r+q}\textbf{I}. \end{aligned}$$

This reduces (8) to the scaler coefficient version (2), with $m=\frac{r}{r+q}$ being the *optimal* momentum coefficient. Because the variation of the key parameters is expected to be much less than that of the query’s, we have $\frac{q}{r}\ll 1$, resulting in $m\simeq 1$, which has been noticed in^1^ experimentally.

## Experimental results

The results of experiments performed on IN-1M, IG-1B, and OpenfMRI are presented below.

### Results on IN-1M

We have evaluated the performance of KalCo and scaler KalCo learning in the same scenarios as those in^1^, under the ImageNet linear classification protocol. We adopt the same pretext task of *instance discrimination* in^3^, and only vary the contrastive loss mechanism. The matrices $\textbf{F}$, $\textbf{Q}$, $\textbf{H}$, and $\textbf{R}$ in KalCo are inferred at each batch from the previous mini-batches through a minimum $\ell _2$-norm criteria for the corresponding $\varvec{\uptheta }_\text {k}$s and $\varvec{\uptheta }_\text {q}$s. The noise variances *q* and *r* in scaler KalCo have also been inferred as such. The number of negatives is *K* = 2^*k*^ in memory bank, MoCo, and KalCos, and is $K-1$ in end-to-end (offset by one because the positive key is in the same mini-batch). The network is ResNet-50. The extra computational load and memory usage for updating the above 4 Kalman matrices are far less than those of this ResNet.

The results are shown in Table 1. Scaler KalCo performs about 10–12% more accurate than MoCo, depending on *K*. Matrix KalCo surpasses MoCo by the even more impressive amounts of 22–25%.

**Table 1:** Accuracies (%) of five contrastive loss mechanisms for $K=2^k$ (*k*=8, 9, 10, 12, 14, 16), in the standard pretext task of instance discrimination on IN-1M.

	$k=8$	$k=9$	$k=10$	$k=12$	$k=14$	$k=16$
KalCo	79.8	81.0	81.5	82.1	82.9	83.0
Scaler KalCo	66.7	68.2	69.0	70.1	70.5	70.6
MoCo	54.7	56.4	57.5	59	60.4	60.6
Memory bank	54.9	56.3	57.3			
End-to-end	50.0	52.0	54.1	56.5	57.8	58.0

### Results on IG-1B

We have also performed our tests on IG-1B. This is a dataset of $\sim$1 billion (940M) public images from Instagram, corresponding to $\sim$1500 hashtags related to the ImageNet categories. It is relatively uncurated compared to IN-1M, and has a long-tailed, unbalanced distribution of real-world data^1^. It also contains both iconic objects and scene-level images. Our tests on this dataset demonstrate accuracies comparable to those in Table 1 (about 98.2% of the values there, on average) as well.

### Results on OpenfMRI

fMRI images are measurements of the blood oxygen level dependent (BOLD) signal, which reflects the changes in blood flow and oxygen consumption in the brain due to neural activity. Because of the good time- and space-resolution of these images, Kalman dynamics can be used to model the relationship between the neural activity and the BOLD signal, as well as the temporal evolution of each. It can also be used to estimate the hidden neural activity from the observed BOLD signal, and to reconstruct or denoise the fMRI images. Paper^8^; e.g., proposes a state space model based on piecewise-linear recurrent neural networks to capture the dynamical changes and fluctuations in brain signals and regions over time. The model is trained using a variation of the Kalman filter and smoother, and applied to simulated and real fMRI data. The work^9^ also presents a neural network architecture that learns from data to carry out Kalman filtering under non-linear dynamics with partial information. It demonstrates the effectiveness of the method on synthetic and real-world data, including fMRI, as well.

We have also performed the instance discrimination task experiment on a bunch of datasets from OpenfMRI. This is a collection of human brain imaging data collected using MRI and EEG techniques. It contains data from various domains and modalities such as vision, language, memory, emotion, and more. The data are organized according to the Brain Imaging Data Structure (BIDS) standard, which ensures a consistent and transparent file naming and metadata scheme across all datasets. It also provides analysis tools and code to facilitate the processing and interpretation of the data. It currently hosts 95 datasets with a total of 3372 subjects. The results are presented in Table 2.

**Table 2:** Accuracies (%) of five contrastive loss mechanisms for $K=2^k$ (*k*=8, 9, 10, 12, 14, 16), in the instance discrimination task on the OpenfMRI datasets.

	$k=8$	$k=9$	$k=10$	$k=12$	$k=14$	$k=16$
KalCo	83.8	85.0	85.6	86.2	87.0	87.1
Scaler KalCo	70.0	71.6	72.4	73.6	74.0	74.1
MoCo	58.0	59.8	61.0	62.5	64.0	64.2
Memory bank	58.2	59.7	60.7			
End-to-end	53.0	55.1	57.3	59.9	61.2	61.5

## KalCo v2

Moco v2^7^ is realized by simple modifications on MoCo v1, consisting of using an MLP projection head and more data augmentation. It can establish stronger baselines that outperform SimCLR, and does not require large training batches. Paper^7^ does not however present any theoretical explanation about why the blur augmentation it performs contributes to such improvements. This has been done below, along also with evaluation of the counterpart KalCo v2.

### Interpretation of the blur augmentation

The blur augmentation used in^7^ can be interpreted as a counterpart to regularized methods for solving optimization problems in ill-posed conditions. Such regularizations improve the generalizability of the model by constraining it at the training time in a way that reflects, in Bayesian regression terms, the prior knowledge about the problem, e.g. by providing information about correlations between features. Similarly, the blur augmentation constructs views from additional domain knowledge in the data. These include; e.g., the level of correlation between each pixel and the other ones in its neighborhood covered by the blurring filter.

This is much like how regularized least squares in linear regression can be viewed as likelihood maximization under an assumption of normally distributed residuals. The regularization terms can then be thought of as actually encoding the further available priors, which lead to the improved techniques of Tikhonov regularization, Lasso regression, $|\ell _0|$ penalization, elastic nets, and total variation regularization.

This interpretation also well explains the non-monotonic relation of linear classification accuracy to transfer performance in detection, hinted at in^7^.

### Experimental results on KalCo v2

We arrange the same experimentation setting as the one in^7^. The *fc* head in MoCo is replaced with a 2-layer MLP head (hidden layer 2048-d, with ReLU). The augmentation used is the extension of the original one in^1^ with the blur augmentation in^4^. For the case of $k=16$ in Table 1, this improves the $83\%$ accuracy of KalCo to 90.4% on ImageNet, and 87.1% to 94.6% on the OpenfMRI datasets, which are indeed remarkable.

#### Comparison with three most-cited recent alternatives

We also compare KalCo v2 with masked auto-encoding (MAE) using ViT-Huge, SimMIM, and Dino v2.

**MAE using ViT-Huge**. Paper^10^ introduces a self-supervised learning framework using masked auto-encoders for computer vision tasks. This framework involves an asymmetric encoder-decoder architecture where the encoder processes only visible patches of an image, and the lightweight decoder reconstructs the entire image from latent representations and masked tokens. A meaningful self-supervision is performed by masking a substantial portion (up to 75%) of the input image. Experimental results demonstrate that this improves training efficiency significantly, achieving a 3$\times$ speedup, along also with an accuracy of 87.8% on ImageNet-1K classification task using a ViT-Huge vision transformer model. MAE outperforms traditional supervised pre-training methods, showcasing excellent transferability to downstream tasks, which validates its scalability and effectiveness in learning high–capacity models that generalize well across various applications.

**SimMIM**. Paper^11^ presents a straightforward approach to masked image modeling (MIM) that simplifies the related existing methods. It randomly masks a significant portion of the input image, specifically using a moderately large patch size (e.g., 32), and predicts the RGB values of the masked pixels by direct regression (rather than relying on more complex alternatives such as patch classification). The authors analyze the components of their framework systematically, and figure out that a lightweight prediction head, such as a linear layer, performs comparably to heavier alternatives. Using a ViT–B, SimMIM achieves a Top–1 fine–tuning accuracy of 83.8% on ImageNet-1K, surpassing the previous best method by 0.6%. Additionally, when applied to a larger model, SwinV2–H, with approximately 650 million parameters, it reaches 87.1% Top–1 accuracy on the same dataset. The method also effectively addresses the data–hungriness drawback of large–scale model training, as demonstrated by training a 3 billion parameter model (SwinV2–G) to achieve state–of–the–art accuracies on the four vision benchmarks of ImageNet–1K, ADE20K, COCO, and JFT-3B, using 40 times less labeled data than previous practices.

**Dino v2**. Paper^12^ introduces another self–supervised learning method for visual feature extraction applied on ViTs. It consists of a two–stage process: first, a teacher model is trained using contrastive learning to generate high–quality visual representations, and then a student model learns from these representations through a distillation process. This allows the model to leverage the strengths of both contrastive and non–contrastive approaches. Dino v2 achieves a Top–1 accuracy of 86.5% on the ImageNet classification task.   ■

We see that the 90.4% accuracy of KalCo v2 on ImageNet is higher than those of all the above three.

## Data Availability

IN–1M data available from ImageNet (https://www.image-net.org), IG–1B from https://paperswithcode.com/dataset/ig-1b-targeted, and fMRI data from OpenfMRI (https://openfmri.org).
